# The Effectiveness of Preemptive Thoracic Epidural Analgesia in Thoracic Surgery

**DOI:** 10.1155/2014/673682

**Published:** 2014-03-13

**Authors:** Engin Erturk, Ferdane Aydogdu Kaya, Dilek Kutanis, Ahmet Besir, Ali Akdogan, Sükran Geze, Ersagun Tugcugil

**Affiliations:** ^1^Department of Anesthesiology and Intensive Care, Faculty of Medicine, Karadeniz Technical University, 61080 Trabzon, Turkey; ^2^Department of Anesthesiology and Intensive Care, Faculty of Medicine, Recep Tayyip Erdoğan University, 53100 Rize, Turkey

## Abstract

*Background*. The aim of this study is to investigate the effectiveness of preemptive thoracic epidural analgesia (TEA) comparing conventional postoperative epidural analgesia on thoracotomy.* Material and Methods*. Forty-four patients were randomized in to two groups (preemptive: Group P, control: Group C). Epidural catheter was inserted in all patients preoperatively. In Group P, epidural analgesic solution was administered as a bolus before the surgical incision and was continued until the end of the surgery. Postoperative patient controlled epidural analgesia infusion pumps were prepared for all patients. Respiratory rates (RR) were recorded. Patient's analgesia was evaluated with visual analog scale at rest (VASr) and coughing (VASc). Number of patient's demands from the pump, pump's delivery, and additional analgesic requirement were also recorded.* Results*. RR in Group C was higher than in Group P at postoperative 1st and 2nd hours. Both VASr and VASc scores in Group P were lower than in Group C at postoperative 1st, 2nd, and 4th hours. Patient's demand and pump's delivery count for bolus dose in Group P were lower than in Group C in all measurement times. Total analgesic requirements on postoperative 1st and 24th hours in Group P were lower than in Group C.* Conclusion*. We consider that preemptive TEA may offer better analgesia after thoracotomy.

## 1. Introduction

Postoperative pain is one of the most important factors affecting the patient's morbidity. Thoracotomy is considered as one of the most severe acute postoperative painful surgeries [1]. Acute pain in these procedures can lead to respiratory and cardiovascular complications [2–4]. Coughing and clearance of secretion can be impaired after thoracotomy in patients with inadequate analgesia. This condition may prolong hospital stay and delay discharge from hospital with increase of the cost. For this reason different analgesic methods such as thoracic epidural analgesia (TEA), paravertebral blocks, and systemic analgesic can be used considered for this purpose. TEA is often regarded as to be the gold standard [5]. It was demonstrated that TEA provided better analgesia than conventional analgesia models in postthoracotomy pain [6–8]. Suitable planned TEA decreases postoperative morbidity and mortality providing optimal analgesia without respiratory insufficiency [9].

Also preemptive analgesia is a concept that a pain therapy is more effective if given before the surgical incision and noxious stimulus [10, 11]. It is thought to decrease the incidence of hyperalgesia and allodynia by decreasing the altered central sensory processing [12]. Therefore, systemic opioid-nonopioid analgesic use (iv, im), local anesthetic infiltration, and epidural or spinal local anesthetic administration have been used for preemptive analgesia [11, 13, 14].

The aim of this study is to find out whether preoperative initiation of epidural analgesia is superior compared to postoperative initiation on postthoracotomy pain.

## 2. Material and Methods

After obtaining the ethics committee approval and patient informed consent 44 patients between the ages of 18 and 65 with ASA I–III risk group have been taken in this study. Sealed envelope method was used for randomization and the patients undergoing elective unilateral thoracotomy operation were divided into two groups (preemptive: Group P, *n* = 22 and control: Group C, *n* = 22). Patients with ASA score of IV or more, body mass index 30 kg/m^2^ or more, and severe renal, hepatic, or neurologic diseases and those using opioid or systemic analgesic preoperatively were excluded from the study.

All patients were administered midazolam 3 mg intramuscularly 30 min before the operation for sedation. In the operating room, electrocardiography, peripheral arterial oxygen saturation, and invasive arterial blood pressure were monitored. After the skin disinfection and lidocaine 20 mg administration for local anesthesia, 18G epidural catheter was inserted at T_5-8_ intervertebral spaces in lateral decubitus position. Propofol (1.5–2.5 mg/kg) and fentanyl (2 *μ*g/kg) were used intravenously for induction anesthesia. After the administration of 0.15 mg/kg of cisatracurium, patients were intubated with double lumen tubes. For anesthesia maintenance, total intravenous anesthesia was used and 125–250 *μ*g/kg/min of propofol with 0.1–0.25 *μ*g/kg/min of remifentanil infusions was started intravenously.

Analgesic solution was prepared for epidural infusion. The solution contained 0.1% levobupivacaine and 2 *μ*g/mL of fentanyl. For patients in preemptive group (Group P) 0.1 mL/kg of bolus standard epidural solution was administered 20 min before surgical incision via epidural catheter. Epidural infusion with 10 mL/h of the same solution was started 45 min after the bolus dose. And it was continued during the operation via epidural catheter. In patients in control group (Group C) equal volume of serum physiologic was administered as a bolus and infusion via epidural catheter during the operation. 0.1 mL/kg of standard epidural solution (0.1% levobupivacaine, 2 *μ*g/mL fentanyl) was administered as a bolus via epidural catheter 20 min before the patient woke up.

After the patients were extubated and all of the drug infusions were discontinued, all patients were transferred to postanesthesia care unit for 24 hours under constant monitoring and clinical observation. Patient controlled epidural analgesia (PCEA) infusor (Abbott Laboratories) was performed on all patients. The PCEA pumps were set as a 5 mL/h of infusion, 3 mL of bolus dose, and 30 min of lock out time. Patient's analgesia was evaluated with visual analog scale (VAS) (0, no pain at all; 10, worst imaginable pain). If the VAS score at rest was 4 or more tramadol 50 mg, as an additional analgesic, was administered intramuscularly.

VAS score at rest (VASr) and on coughing (VASc) and demand and delivery count of PCEA infusor were independently measured at postoperative 1st, 2nd, 4th, 6th, 12th, and 24th hours by a trained physician blinded to the randomization. Total tramadol requirements at postoperative 1st and 24th hour were recorded. The incidence of side effects such as nausea, vomiting, and pruritus was also recorded. Mean arterial pressure (MAP), heart rate (HR), and respiratory rate (RR) were measured at the same time periods. Hypotension was defined as a decrease in mean arterial pressure below 60 mmHg lasting at least 30 min and bradypnoea was defined as a respiratory rate <10 bpm. It was planned that the patient who developed hypotension or bradypnoea was treated and excluded from the study.

### 2.1. Statistical Analysis

Data were presented in the form of mean ± SD. All statistical analyses were carried out using SPSS statistical software (SPSS for windows, version 14.0). The Kolmogorov-Smirnov test was used to determine normality and homogeneity of data distribution. Parametric data (age, blood pressure, and OLV time) were compared using one-way analysis of variation (ANOVA). Nonparametric data were compared using the Kruskal-Wallis test. Mann-Whitney *U* test was for pain scores.

## 3. Results

There were no significant differences between the groups with respect to age, sex, ASA score, and surgery time (Table 1). Although MAP and HR were insignificant in comparison of the groups, RR in Group C was higher than in Group P at postoperative 1st and 2nd hours (postoperative 1st hour: 21.05 ± 3.72, 18.25 ± 3.64, postoperative 2nd hour: 20.45 ± 3.41, 18.35 ± 2.83, resp.) (*P* < 0.05) (Table 2).

Data on postoperative pain at rest (VASr) and coughing (VASc) are shown in Tables 3 and 4. Both VASr and VASc scores in Group P were lower than in Group C at postoperative 1st, 2nd, and 4th hours (*P* < 0.01) (Tables 3 and 4).

**Figure 1:**
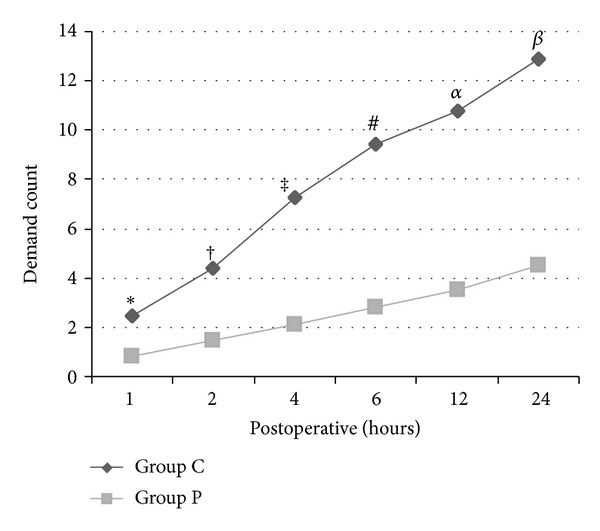
Patient's demand count on PCEA pump when Group C is compared to Group P (*: *P* = 0.013, ^†^: *P* = 0.000, ^‡^: *P* = 0.002, ^#^: *P* = 0.001, ^*α*^: *P* = 0.000, and ^*β*^: *P* = 0.000).

**Figure 2:**
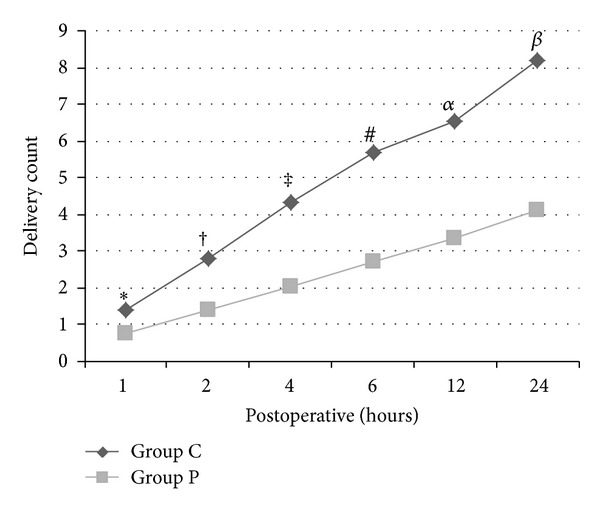
Pump's delivery count on PCEA pump when Group C is compared to Group P (*: *P* = 0.013, ^†^: *P* = 0.000, ^‡^: *P* = 0.002, ^#^: *P* = 0.001, ^*α*^: *P* = 0.000, and ^*β*^: *P* = 0.000).

When PCEA pump was examined, patient's demand and pump's delivery count for bolus dose in Group P were lower than in Group C on all measurement times (*P* < 0.01) (Figures 1 and 2).

**Figure 3:**
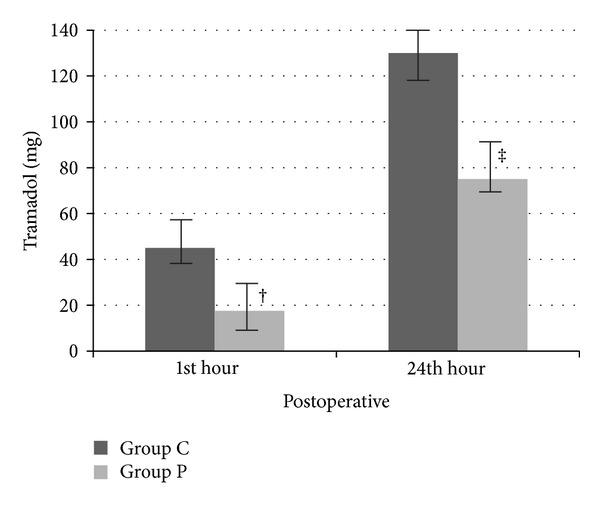
Total analgesic requirement. ^†^: *P* = 0.004 when tramadol amount at 1st postoperative hour in Group C was compared with those in Group P. ^‡^: *P* = 0.032 when tramadol amount at 24th postoperative hour in Group C was compared with those in Group P.

When the additional analgesic requirement was compared, total tramadol amount on postoperative 1st and 24th hours in Group P was lower than in Group C (postoperative 1st hour: 17.5 ± 14.4, 45.0 ± 22.3, postoperative 24th hour: 75.0 ± 63.8, 130.0 ± 89.4, resp.) (*P* < 0.01 and *P* < 0.05, resp.) (Figure 3).

There were no differences between the groups with respect to side effects.

## 4. Discussion 

This study showed that preincisional epidural initiation provided better analgesia than postoperative application for postthoracotomy pain. Pain score at rest and coughing were lower with preincisional initiation, especially in early postoperative period. Decreased number of patient's demand from PCEA pump and pump's delivery to the patients in preemptive group supported the idea that preemptive analgesic initiation was superior compared to postoperative initiation.

Previous studies were carried out to find out the benefit of preemptive analgesia. Bong et al. [1] stated that the effectiveness of preemptive epidural analgesia is more clear in thoracotomy surgery than in other surgical procedures. It was stated that thoracotomy produces excessive noxious stimuli caused by central sensitization [15–17]. Hence we carried out this study in patients undergoing thoracic surgery to demonstrate the effectiveness of preemptive epidural analgesia.

Yegin et al. [18] investigated the effectiveness of pre- and postoperative epidural analgesia versus postoperative epidural analgesia in thoracic surgery. They administered bupivacaine and fentanyl as a bolus to intervention group preoperatively. PCEA was applied to each group with the same protocol and VAS scores were recorded postoperatively. They found better analgesia with the preoperative initiation of epidural analgesia. Their findings were similar to our results.

Amr et al. [19] carried out a study to find out the effects of preincisional epidural application on pulmonary and endocrine system besides pain. They showed significant improvement in pulmonary functions along with better analgesia in preincisional group as compared with the postoperative group. However, the oxygenation, cortisol, or glucose levels were found insignificant and concluded that, although preemptive analgesia provided better analgesia and preserved pulmonary functions, it had no effect on stress response and these findings were not enough to conclude a clinical significant difference. The amount of epidural local anesthetic may lead to this indifference between the groups on stress response. Their patient's VAS score at rest and coughing was relatively high (VAS > 3 in early postoperative period). Motor block is most fearful complication of thoracic epidural analgesia with local anesthetic. As they used bupivacaine, powerful motor blocking agent, they could not administer more high dose epidural local anesthetic. If better analgesia was provided, the positive effect on stress response might be demonstrated.

Ideal local anesthetic agent for thoracic epidural analgesia must have fast and long acting analgesia, lower motor block and hemodynamic side effects, and higher toxic dose limit [20]. Levobupivacaine, S-enantiomer of racemic bupivacaine, is along acting local anesthetic that caused less neuro- and cardiotoxic side effects than other local anesthetics [9, 21]. These properties of levobupivacaine enable it to be used in higher doses safely to achieve sufficient analgesia. Thus, we chose levobupivacaine and achieved required analgesia. Mendola et al. [22] used 10 mg/h levobupivacaine via epidural catheter postoperatively for postthoracotomy pain and stated that this application can provide sufficient analgesia. However, they administered proparacetamol 1.5 gr and ketorolac 60 mg daily to patients. If the epidural levobupivacaine initiated preoperatively, these analgesics were not needed.

Chronic postthoracotomy pain is recurred or persisted along the thoracotomy scar more than two months after surgery [23]. It was stated that acute pain after thoracotomy was related to chronic postthoracotomy pain [17]. Thus, studies were carried out to demonstrate the preventive effects of preemptive epidural analgesia on chronic postthoracotomy pain [24–27]. Şentürk et al. [25] compared the effects of TEA with and without preoperative initiation on postthoracotomy pain. At the end of their study, they stated that TEA with preoperative initiation can prevent acute and long term thoracotomy pain. Similarly, Ochroch et al. [26] carried out a study, but with higher dose of bupivacaine, to investigate the effectiveness of preemptive TEA. They concluded a benefit of preemptive analgesia.

On the other hand, studies show that clinical effectiveness of preemptive analgesia is controversial [28–30]. Neustein et al. [27] compared the pre- versus postoperative initiated TEA using bupivacaine. They found that preemptive TEA provided better analgesia until postoperative 6th hour and VAS scores after 6th hour which is insignificant. We also found similar findings. But their VAS scores in both pre- and postoperative initiation groups were higher than ours. They used only preoperative bolus of bupivacaine, but not infusion. Higher VAS scores may be explained by insufficient analgesia.

Although our findings encourage us to use preemptive TEA to provide sufficient analgesia after thoracotomy there were some limitations in our study. We record VAS scores only until postoperative 24th hour. And we did not investigate the effects of TEA on pulmonary functions and stress response in more detail. If we had evaluated these parameters, this study may be more powerful.

In conclusion we consider that preemptive TEA may offer better analgesia after thoracic surgery. However, further studies with more patients are needed to demonstrate the benefits of preemptive epidural analgesia providing better analgesia with less side effects and positive outcomes from stress response.

## Figures and Tables

**Table 1:** Patients characteristic and surgery time.

	Group C	Group P
Age (years)	52.35 ± 13.38	51.75 ± 13.70
Sex (M/F)	14/8	15/7
ASA (I/II/III)	2/12/8	4/11/7
Surgery time (hours)	3.05 ± 1.02	3.25 ± 0.91

**Table 2:** Mean arterial pressure (MAP; mmHg), heart rate (HR; beat/min), and respiratory rate (RR; count/min).

	Group C			Group P		
	MAP	HR	RR	MAP	HR	RR
Postoperative 1st hour	67.35 ± 12.30	87.40 ± 19.30	21.05 ± 3.72*	66.45 ± 11.20	83.30 ± 11.31	18.25 ± 3.64
Postoperative 2nd hour	66.25 ± 11.30	86.45 ± 16.40	20.45 ± 3.41*	64.20 ± 12.90	79.90 ± 12.36	18.35 ± 2.83
Postoperative 4th hour	66.12 ± 13.90	84.00 ± 13.60	19.35 ± 3.76	66.12 ± 13.90	82.10 ± 12.50	17.90 ± 3.09
Postoperative 6th hour	68.15 ± 12.86	83.05 ± 11.82	18.65 ± 2.51	66.15 ± 14.55	84.20 ± 12.06	18.25 ± 3.12
Postoperative 12th hour	66.55 ± 14.11	83.65 ± 11.01	18.50 ± 2.70	66.65 ± 12.00	82.50 ± 13.08	18.05 ± 2.58
Postoperative 24th hour	67.10 ± 12.99	84.00 ± 13.39	18.80 ± 2.16	65.60 ± 12.00	82.05 ± 8.06	19.00 ± 2.92

*P < 0.05 when RR at 1st and 2nd postoperative hours in Group C was compared with those in Group P.

**Table 3:** Postoperative pain score at rest (VASr) (mean ± SD).

	Group C	Group P	*P* value
Postoperative 1st hour	4.05 ± 2.18^*β*^	1.90 ± 1.21	**0.002**
Postoperative 2nd hour	3.45 ± 2.23^*α*^	1.40 ± 0.94	**0.001**
Postoperative 4th hour	2.60 ± 1.93*	1.20 ± 0.83	**0.009**
Postoperative 6th hour	1.45 ± 1.27	1.05 ± 1.63	0.134
Postoperative 12th hour	1.10 ± 1.37	0.50 ± 0.82	0.134
Postoperative 24th hour	0.75 ± 1.02	0.35 ± 0.81	0.192

^*β*^When VASr scores at 1st postoperative hour in Group C were compared with those in Group P.

^*α*^When VASr scores at 2nd postoperative hour in Group C were compared with those in Group P.

*When VASr scores at 4th postoperative hour in Group C were compared with those in Group P.

**Table 4:** Postoperative pain score at coughing (VASc) (mean ± SD).

	Group C	Group P	*P* value
Postoperative 1st hour	4.95 ± 2.01^*β*^	3.15 ± 1.22	**0.007**
Postoperative 2nd hour	4.40 ± 2.08^*α*^	2.55 ± 1.14	**0.004**
Postoperative 4th hour	3.50 ± 1.76*	2.20 ± 0.95	**0.009**
Postoperative 6th hour	2.55 ± 1.31	2.10 ± 1.48	0.192
Postoperative 12th hour	2.20 ± 1.54	1.40 ± 0.94	0.108
Postoperative 24th hour	1.60 ± 1.18	1.05 ± 1.05	0.121

^*β*^When VASr scores at 1st postoperative hour in Group C were compared with those in Group P.

^*α*^When VASr scores at 2nd postoperative hour in Group C were compared with those in Group P.

*When VASr scores at 4th postoperative hour in Group C were compared with those in Group P.
